# Assessing treatment response in multiple sclerosis

**DOI:** 10.1097/WCO.0000000000001493

**Published:** 2026-04-07

**Authors:** Fabian Föttinger, Nik Krajnc, Gabriel Bsteh

**Affiliations:** a Department of Neurology; b Comprehensive Center for Clinical Neurosciences and Mental Health, Medical University of Vienna, Vienna, Austria

**Keywords:** disease-modifying treatment, multiple sclerosis, outcome, treatment response

## Abstract

**Purpose of the review:**

The evaluation of treatment response in multiple sclerosis (MS) has become increasingly nuanced as the field shifts from a primarily relapse-oriented perspective toward a more comprehensive understanding of the continuous interaction between inflammatory and neurodegenerative mechanisms.

**Recent findings:**

Traditional clinical measures, while indispensable, capture only a fraction of the disease process and are insufficient to detect the insidious progression that accounts for a substantial proportion of long-term disability. Enhanced functional assessments provide greater sensitivity to subtle clinical deterioration, and MRI remains the principal modality for identifying subclinical focal inflammatory activity. In parallel, biomarkers of neurodegeneration, including inner retinal layer thinning measured by optical coherence tomography (OCT) and fluid biomarkers such as serum neurofilament light chain (sNfL) and glial fibrillary acidic protein (GFAP), offer complementary insights into diffuse pathological processes that elude conventional monitoring.

**Summary:**

A personalized monitoring strategy that integrates clinical assessment, imaging and fluid biomarkers holds the greatest promise for improving the detection of subclinical disease activity, refining risk stratification, and enabling more informed and individualized treatment decisions in contemporary MS care.

## INTRODUCTION

Evaluating whether treatment in multiple sclerosis (MS) achieves sufficient disease control remains a major clinical challenge, largely due to the simultaneous presence of inflammatory and neurodegenerative processes that give rise to highly heterogeneous disease trajectories [1,2]. With the growing availability of high-efficacy disease-modifying therapies (DMT), the evolution of therapeutic paradigms, and the emergence of novel imaging and fluid biomarkers, assessing treatment response has become increasingly complex [1,3^▪▪^]. In current practice, therapeutic effectiveness is inferred from a synthesis of clinical outcomes and paraclinical measures designed to capture both focal inflammatory activity and disability progression, traditionally summarized under the term “no evidence of disease activity” (NEDA) [4]. Historically, NEDA has developed as a post-hoc endpoint in clinical trials and is now most commonly operationalized as a three-component framework, defined by the absence of clinical relapses, confirmed disability worsening, and MRI-detected disease activity (NEDA-3) [4]. While NEDA provides a practical and widely adopted framework, it mainly reflects control of overt inflammatory events and is less sensitive to the insidious processes that drive long-term disability [5,6]. Consequently, treatment evaluation is shifting toward frameworks that better account for progression occurring independently of relapses [7].

This recognition is fueling a transition toward a personalized treatment paradigm in which clinical assessment is complemented by sensitive biomarkers capable of detecting subclinical neuroaxonal injury. Inner retinal layer thinning as measured by optical coherence tomography (OCT) and fluid biomarkers such as serum neurofilament light chain (sNfL) and glial fibrillary acidic protein (GFAP) levels offer concrete and biologically grounded markers of neurodegeneration and treatment responsiveness. When combined with established clinical metrics, these tools have the potential to enhance individualized monitoring, enable earlier identification of treatment insufficiency, and capture dimensions of disease evolution that elude conventional assessments.

Against this backdrop, we offer a structured overview of the principal clinical and paraclinical measures used to evaluate treatment response in MS, with the aim of outlining a more comprehensive and biologically informed framework for monitoring treatment response. 

**Box 1:**
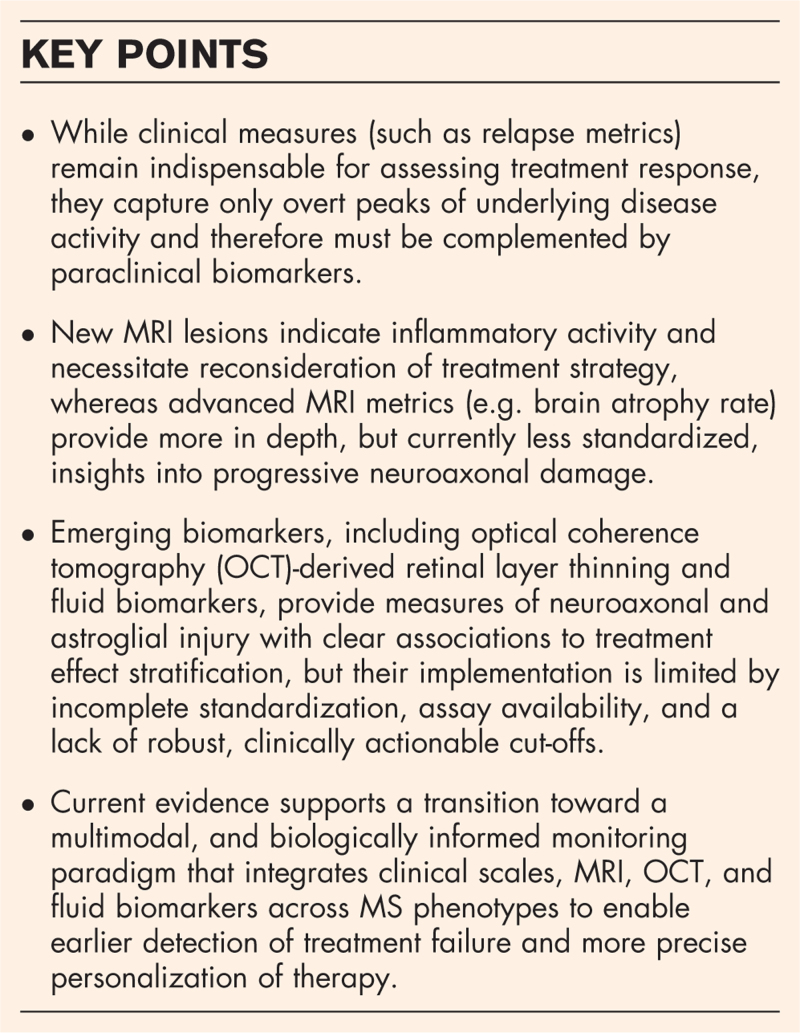
no caption available

## CLINICAL TREATMENT RESPONSE MONITORING

Clinical evaluation remains the cornerstone of assessing treatment response in MS, encompassing both transient and persistent neurologic impairments. This evaluation traditionally relies on monitoring relapse occurrence, quantifying neurological disability via the Expanded Disability Status Scale (EDSS), and/or measuring objective clinical performance across functional domains [8].

### Relapse and relapse-free status

**FIGURE 1:**
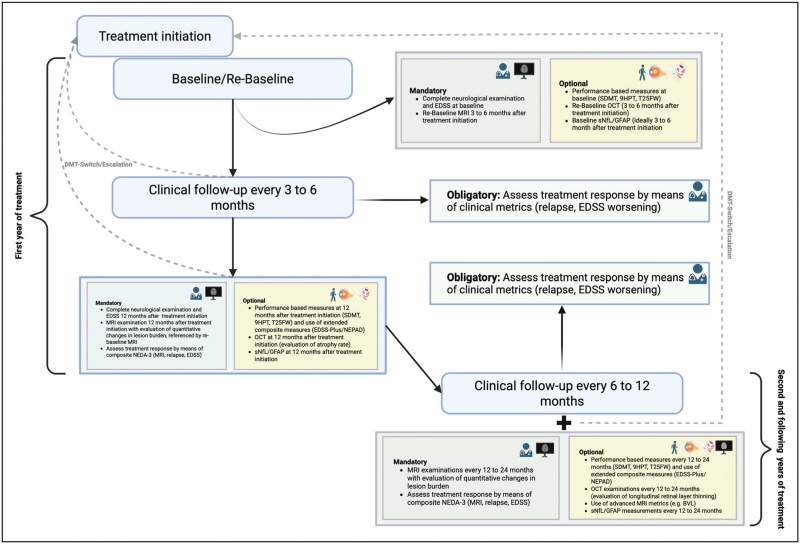
Schematic overview of treatment monitoring algorithm. 9HPT, 9-hole peg test; DMT, disease-modifying treatment; EDSS, Expanded Disability Status Scale; GFAP, glial fibrillary acidic protein; MRI, magnetic resonance imaging; NEDA, no evidence of disease activity; NEPAD, no evidence of progression or active disease; OCT, optical coherence tomography; SDMT, Symbol Digit Modalities Test; sNfL, serum neurofilament light chain; T25FW, timed 25-foot walk.

Relapses are characterized by the acute onset of new neurologic deficits and/or exacerbation of preexisting symptoms in the absence of fever or infection, and represent the clinical hallmark of focal inflammatory MS pathology [1]. The annualized relapse rate (ARR) continues to serve as the predominant primary endpoint in clinical trials and retains a central role in establishing the anti-inflammatory efficacy of DMT [9^▪^]. In clinical practice, achieving relapse-free status remains a key therapeutic objective in relapsing MS (RMS) cohorts (Fig. 1, Table 1) [10^▪^,^11^]. Any confirmed relapse during treatment signals suboptimal therapeutic response and heightens the risk of persistent neurologic impairment, thereby necessitating timely reassessment of the therapeutic strategy (e.g., evaluation of treatment adherence, escalation, or switching) [5,10^▪^].

**Table 1:** Clinical measures of treatment response

Measure	Type	Domain	Clinically meaningful change	Limitations
EDSS	Clinician-rated scale	Global disability, neurologic impairment rated in functional systems	Operationalized as CDP with no universal definition; Commonly used definition: increased EDSS ≥ 1.5, ≥ 1.0, ≥ 0.5 for baseline EDSS 0, 1–5.5, ≥ 6 persistent for ≥ 24 weeks RAW (CDP within 90 days of a relapse): carries a clear implication to consider treatment escalation PIRA (CDP with no relapse in the 90 days preceding the initial event): incompletely defined implications for treatment sequencing	Non-linear Gait-heavy and insensitive to UEX/cognitive impairment High inter-rater variability
Relapse/ ARR	Clinician-rated event count	Acute focal inflammatory attacks; transient or persistent neurologic deficit	Any objective relapse is clinically meaningful	Poor sensitivity to progressive disease activity (i.e. PIRA) Heterogeneity in relapse ascertainment ARR does not account for relapse severity or residual deficits
T25FW	Performance test	Ambulation speed	A worsening of ≥ 20% in walking speed from baseline; confirmation interval required (typically 3 to 6 months)	Measures short-distance walking speed Considerable short-term variability Floor/ceiling effects (minimal impairment/high impairment)
9HPT	Performance test	Upper limb dexterity (fine motor)	A worsening of ≥20% in 9HPT completion time from baseline; confirmation interval required (typically 3 to 6 months)	Notable ceiling and floor effects (minimal impairment/high impairment)
SDMT	Cognitive performance test	Processing speed, attention	A change of ≥ 4 points (or about 10% of the score) from baseline is typically regarded as a clinically meaningful cognitive change; confirmation interval required (typically 3 to 6 months)	Susceptible to practice effects Sensitive but non-specific indicator of cognitive status

9HPT, 9-hole peg test; ARR, annualized relapse rate; CDP, confirmed disability progression; EDSS, Expanded Disability Status Scale; PIRA, progression independent of relapse activity; SDMT, Symbol Digit Modalities Test; T25FW, timed 25-foot walk; UEX, upper extremity.

Despite the intuitive clinical relevance of relapse monitoring, relapse-based metrics face notable limitations. Relapses occur relatively infrequently, are vulnerable to heterogeneity in ascertainment, and demonstrate substantial rater dependence [10^▪^,^12^]. Their sensitivity for capturing overall disease activity is low, as roughly one clinical relapse occurs for every ten new MRI lesions and the minimal detectable change equals the threshold for clinically meaningful change (i.e., the occurrence of a relapse) [10^▪^]. Furthermore, relapse frequencies are inherently less informative in progressive MS (PMS) and only partially account for long-term disability accumulation. A considerable proportion of worsening arises from relapse independent mechanisms, even among individuals with relapsing phenotypes [5,9^▪^,^13^]. This was underscored in a recent Italian MS Registry analysis with ≥5 years of follow-up, where 72.3% of all confirmed disability accrual occurred independent of relapse activity [14]. Collectively, these data emphasize that although relapse metrics remain clinically meaningful, they are insufficient as standalone surrogates of treatment response.

### Expanded disability status scale

The Expanded Disability Status Scale (EDSS) remains a cornerstone outcome measure in MS clinical trials and regulatory evaluations, supported by decades of use and broad acceptance as an index of disability [4,15]. Derived from a structured neurological examination as originally defined by Kurtzke *et al.*, the EDSS integrates eight functional systems (pyramidal, cerebellar, brainstem, sensory, visual, bowel/bladder, cerebral/mental, and others) along with graded assessments of ambulation to generate an ordinal score from 0 (normal neurological examination) to 10 (death due to MS) [16]. Its ubiquitous use, easy interpretability, and regulatory familiarity also contribute to its feasibility in everyday clinical practice (Table 1) [15]. Contemporary data continue to affirm the clinical meaningfulness of the EDSS, demonstrating that higher scores correlate with increased risk of future disability progression, reduced quality of life, and elevated healthcare utilization and caregiver burden [17].

In the context of ongoing treatment with DMT, sustained EDSS worsening is interpreted as a marker of inadequate therapeutic response. Accordingly, modern DMT trials typically define confirmed disability progression (CDP) as a baseline-dependent increase in EDSS that persists at 12–24 weeks, with 24-week confirmation increasingly preferred for its superior specificity and robustness. In the context of CDP, two distinct patterns have been observed: progression occurring by incomplete remission of a relapse [relapse-associated worsening (RAW)] or independent of relapse activity [i.e. progression independent of relapse activity (PIRA)]. RAW is thought to represent the clinical correlate of disability worsening through overt focal inflammatory activity, whereas PIRA is suggested to be linked to insidious inflammation causing neurodegenerative processes [3^▪▪^]. Differentiation is important in daily clinical practice, as DMT primarily target inflammation and accumulating evidence suggests a more limited effect of DMT on PIRA events compared to RAW. In the OPERA trials, ocrelizumab reduced the risk of RAW by 47% compared to interferon beta, but only by 22% for PIRA [13]. Similarly, real-world data show that patients under DMT have fewer relapses and delayed progression overall, yet a higher relative proportion of their worsening occurs through PIRA [5]. Thus, PIRA is evidently a sign of improper treatment response, however its precise role in guiding treatment sequencing within the current DMT landscape remains to be fully elucidated.

Furthermore, progression rates diverge markedly across studies due to heterogeneity in baseline handling, confirmation intervals, and thresholds, which constrains direct comparability [6,10^▪^,^18^]. Recent narrative and methodological reviews highlight these definitional sensitivities, particularly in PMS cohorts (with progression rates ranging from 28% to 82% over 2–3 years depending on the definition) [19]. Additionally the EDSS is non-linear (implying that a 1-point change does not represent the same disability increment across the scale) and is heavily weighted toward ambulation, which underrepresents upper-limb and cognitive dysfunction [20]. These features diminish sensitivity to clinically relevant change, especially in individuals at higher disability levels or in whom progression manifests outside gait.

Inter-rater variability poses an additional concern, particularly in real-world settings [18]. However, targeted standardization improves reliability, as a randomized, multicenter study reported high concordance (0.87; 95% CI 0.815–0.925) between trained health-care professionals and Neurostatus-certified neurologists, supporting the premise of a decentralized and scalable EDSS acquisition with proper training [21^▪^]. While the EDSS continues to serve as a foundational metric of treatment response, interpretation of longitudinal change must account for its limitations and should be complemented by additional measures that capture domains underrepresented by the EDSS (e.g., cognition) [6].

### Performance-based clinical measures

Performance-based measures provide complementary, domain-specific insights into functional change and are increasingly integrated into both research and clinical practice. The most widely used instruments include the Symbol Digit Modalities Test (SDMT) for cognitive information-processing speed, the Timed 25-Foot Walk (T25FW) for gait and ambulation, and the 9-Hole Peg Test (9HPT) for upper-limb dexterity (Fig. 1, Table 1).

The SDMT correlates strongly with vocational outcomes and everyday functioning, with a 4-point change frequently applied as a clinically meaningful benchmark on a group level [22]. However, at the individual-patient level, reliable-change approaches, such as a ≥10% decline from baseline, offer more robust thresholds and effectively accommodate practice effects, particularly when assessments span 6–12-month intervals or involve hybrid in-person/remote testing [23,24].

The T25FW provides a sensitive, easily accessible, objective index of walking speed. A ≥20% worsening from baseline remains a pragmatic and empirically supported benchmark for clinically meaningful decline, with recent work confirming good reliability in MS cohorts [25,26]. Nevertheless, the smallest detectable change scales with baseline performance (≈0.23 × mean T25FW), requiring caution when interpreting small numerical variations among slower baseline walkers [27].

The 9HPT captures manual dexterity, underrepresented in EDSS, with a ≥20% slowing likewise treated as clinically meaningful [28]. While feasible for clinical use, its interpretability is more limited in very mild (EDSS ≤ 3.0) or advanced (EDSS ≥ 6.0) disability ranges due to floor and ceiling effects, and results may vary depending on pegboard type [29].

Importantly, recent analyses of pooled phase III clinical trial datasets demonstrated that worsening on the T25FW or 9HPT predicts a significantly increased risk of subsequent EDSS progression, even among individuals without early EDSS events (T25FW adjusted HR 1.74–3.26; 9HPT adjusted HR 1.45–3.08) [30^▪^]. These findings align with earlier work showing that the inclusion of T25FW/9HPT events in extended CDP definitions nearly doubles the detection of 24-week confirmed progression (≈59.5% vs. 24.7% using EDSS alone) [31].

These performance-based measures have also been combined in the multiple sclerosis functional composite (MSFC), which integrates T25FW, 9HPT, and a cognitive component, now often substituted with the SDMT in lieu of the traditional Paced Auditory Serial Addition Test (PASAT), into a standardized z-score [32]. The MSFC has been thoroughly validated over the last two decades and remains valuable in clinical trials, observational cohorts and routine practice [15,32,33]. In parallel, other composite treatment targets have been proposed, combining performance-based measures, EDSS-defined disability progression and/or other biomarkers of disease activity. For example, the EDSS-Plus is a composite endpoint defined by 24-week confirmed worsening in either the EDSS, T25FW, or 9HPT. The EDSS-Plus identified about 60% of patients with secondary progressive MS (SPMS) as progressive over 2 years (vs. 25% by EDSS alone), indicating markedly increased sensitivity [31]. Similarly, “no evidence of progression or active disease” (NEPAD) extends the concept by requiring no new relapses or MRI lesions in addition to no confirmed worsening on EDSS, T25FW or 9HPT, providing a more sensitive tool particularly in both relapsing and progressive MS populations [34]. Such composite measures may detect disease progression earlier than EDSS alone but are complex and resource-intensive (requiring multiple timed tests and MRI) and have so far been applied mainly in trial settings [34]. Moreover, their utility in informing treatment decisions, including DMT escalation, has not yet been systematically evaluated.

## PARACLINICAL TREATMENT RESPONSE MONITORING

Beyond clinical treatment monitoring, paraclinical biomarkers are essential tools for detecting subtle, subclinical indicators of treatment failure. While MRI remains a well-established and widely accessible modality, recent advances in OCT and fluid biomarkers have expanded the scope of measurable disease activity, offering complementary perspectives on neuroaxonal integrity. These developments mark a significant step toward more biologically sensitive and individualized monitoring strategies.

### Magnetic resonance imaging

MRI has served as an established biomarker for monitoring disease activity in MS for several decades, providing a sensitive tool to detect subclinical inflammatory activity that often occurs independently of overt clinical relapses. Although there is broad consensus that new T2 lesions represent a more sensitive marker of disease activity than clinical measures, the threshold number of new T2 lesions indicative of treatment failure, and thus predictive of long-term prognosis, remains a matter of debate, with proposed cut-offs ranging from a single new T2 lesion to more than three [6,35,36]. More recently, a threshold of more than two new T2 lesions has been suggested as a meaningful indicator of isolated MRI activity, especially in clinically stable patients (Table 2) [37^▪^].

**Table 2:** Paraclinical measurements of treatment response

Measure	Type	Domain	Clinically meaningful change	Limitations
New/enlarging T2-lesions and/or gadolinium-enhancing lesions	MRI	Focal inflammatory MS pathology	≥ 2–3 new/enlarging T2-hyperintense lesions and/or ≥ 1 gadolinium-enhancing lesion [37^▪^]	Moderate sensitivity Inter-rater variability
Brain volume loss	MRI	Subclinical neurodegeneration	≥ 0.4%/year volumetric loss over a multi-year horizon [81]	High technical requirements (hardware and software) Long observation periods Pseudoatrophy Multiple confounding factors (age, comorbidity, comedication, etc.)
Retinal layer thickness (GCIPL/pRNFL)	OCT	Neuro-axonal degeneration; optic nerve pathway	Incompletely defined; Current evidence suggests a prognostically unfavourable factor or insufficient treatment response for: Cross-sectional measurement [82^▪▪^] pRNFL thickness ≤ 88 μm and/or GCIPL ≤ 77 μm Longitudinal measurement [82^▪▪^,^83^] pRNFL thinning of > 1.5 μm/year and/or mGCIPL thinning of ≥ 1.0 μm/year	Rigorous quality control Segmentation error Floor effects Ophthalmologic comorbidities
NfL	Fluid biomarker	Axonal degeneration	Incompletely defined; Current evidence suggests persistently elevated or rising age-adjusted z-scores; however, no cut-off specified	Threshold uncertainty Inter-assay variability Non-specificity Confounding factors (age, BMI, disease activity)
GFAP	Fluid biomarker	Reactive astrogliosis	Incompletely defined; Current evidence suggests persistently elevated or rising age-adjusted z-scores; however, no cut-off specified	Threshold uncertainty Inter-assay variability

GCIPL, ganglion-cell and inner plexiform layer; GFAP, glial fibrillary acidic protein; MRI, magnetic resonance imaging; MS, multiple sclerosis; NfL, neurofilament light chain; pRNFL, peripapillary retinal nerve fiber layer.

While identifying new T2 lesions is characterized by excellent test-retest reliability and minimal interrater variability, evaluating enlarging T2 lesions is considerably more susceptible to measurement variability [10^▪^]. A further challenge lies in interpreting MRI activity during the first year of DMT initiation, when residual pre-treatment inflammatory activity and delayed therapeutic onset may contribute to incident lesions, reflecting a “carry-over” phenomenon rather than true treatment failure [38]. Consequently, current recommendations advise obtaining a re-baseline MRI 3–6 months after treatment initiation to minimize the influence of such effects on subsequent assessments (Fig. 1) [39^▪^].

Gadolinium-enhancing (contrast-enhancing) lesions (CELs), similar to new T2 lesions, capture subclinical disease activity and exhibit excellent test-retest reliability as well as low interrater variability [10^▪^]. In contrast to new T2 lesions, clinically meaningful change in CEL burden appears to be more clearly defined, as the occurrence of even a single CEL is considered indicative of treatment failure [40]. Nevertheless, their utility is limited by the relative infrequency of such events. Moreover, current expert consensus advises against the routine use of gadolinium-based contrast agents in the longitudinal monitoring of patients with MS due to the potential risk of its accumulation in the brain [41].

Whole brain volume, a well-established imaging marker of neurodegeneration, has been inconsistently and only marginally targeted in prior clinical trials, with limited therapeutic success. A change of approximately 0.4% per year is generally regarded as clinically meaningful [42]. Still, whole brain volume measurement is vulnerable to several confounding factors, including hydration status, diurnal variation, lifestyle influences (such as smoking and alcohol consumption), concomitant medications, and comorbidities [10^▪^,^43^,^44^]. Moreover, inflammatory activity can transiently increase brain volume, while DMT may lead to early, non-tissue-related brain volume loss, termed pseudoatrophy, due to the resolution of edema [45]. To mitigate these effects, a re-baseline MRI is recommended 3–6 months after DMT initiation. However, the routine integration of brain atrophy measurement also remains challenging due to technical limitations, including variability in MRI acquisition protocols, scanner-induced distortions, and inter-site inconsistencies, all of which compromise measurement reliability [46]. Owing to its relatively slow rate of change, meaningful assessment typically requires prolonged observation periods, ideally two to three years, rendering it less suitable for clinical practice, where timely treatment adaptation is required.

### Optical coherence tomography

OCT is a non-invasive and readily accessible imaging modality that employs near-infrared light to produce high-resolution images of the retina, enabling precise quantification of the peripapillary retinal nerve fiber layer (pRNFL) and ganglion cell-inner plexiform layer (GCIPL), both of which have emerged as robust and reproducible biomarkers of neuroaxonal degeneration in MS [47,48]. The development of spectral domain OCT (SD-OCT), incorporating advanced eye-tracking systems, automated segmentation algorithms, and frame averaging techniques, has substantially enhanced measurement reliability and sensitivity, allowing for the longitudinal monitoring of subtle micrometer-scale alterations in retinal morphology [49].

Patients failing to achieve NEDA-3 exhibit significantly higher rates of pRNFL thinning over a two-year follow-up period [50]. A threshold of −1.25 μm of pRNFL thinning has been shown to distinguish NEDA from EDA patients with a specificity of 81.4% and a sensitivity of 80% [50]. Although some studies have reported no significant differences in retinal layer atrophy rates between untreated patients and those receiving first-line DMTs over a 1-year period, [51–53] such a time frame may be insufficient to reliably capture therapeutic effects. Longer-term studies have demonstrated that DMT attenuates inner retinal layer thinning, [54–56] consistent with its known capacity to reduce relapse rate and, consequently, axonal injury.

While pRNFL thinning can serve as a marker of treatment failure, it is comparatively less sensitive and typically requires a longer, 24-month interval to achieve acceptable diagnostic precision (specificity 84%, sensitivity 69%) [57]. In contrast, GCIPL thinning offers a more reliable and timely predictor of treatment failure, demonstrating higher specificity and sensitivity (specificity 85%, sensitivity 78%) even over a shorter, 12-month interval, with a modest further improvement in predictive accuracy when extending the observation period to 24 months [57]. Similar to MRI, applying a re-baseline framework to OCT has been shown to substantially improve the differentiation of DMT-specific effects on retinal layer thinning by minimizing carry-over confounding from prior disease activity (Fig. 1) [58]. Moreover, extending the perspective beyond overt neuroinflammatory activity, both PIRA and PIRMA have been shown to be associated with both pRNFL and GCIPL thinning [59,60].

Despite accumulating evidence supporting the clinical utility of OCT in MS, its role in treatment monitoring requires further validation through large-scale prospective studies. Retinal layer thinning, while informative, lacks disease specificity and remains susceptible to confounding by retinal pathology beyond MS and technical limitations, including segmentation errors and flooring effects. Moreover, although emerging evidence is compelling at the group level, caution is warranted when using inner retinal layer thinning to guide treatment escalation in individual patients.

### Fluid biomarkers

#### Neurofilament light chain

NfL, a structural protein of axonal cytoskeleton, is released into the cerebrospinal fluid (CSF) and, subsequently, into the peripheral circulation following neuroaxonal injury, serving as a highly sensitive – yet non-specific – biomarker of axonal damage [61]. Historically, NfL studies were largely restricted to CSF due to the limited sensitivity of earlier detection methodologies. However, the advent of Single Molecule Array (Simoa) technology has revolutionized this landscape, enabling the ultra-sensitive quantification of NfL not only in CSF but also in serum and plasma [62]. Accumulating evidence demonstrates a robust correlation between CSF and serum NfL (sNfL) levels, supporting the application of sNfL as a minimally invasive and readily accessible biomarker for disease monitoring [63]. While sNfL levels are significantly dependent of age and body mass index, these influences on sNfL levels have been mitigated through the use of z-scores, which express the degree of an individual's deviation from reference populations.

Elevated baseline sNfL levels appear to predict subsequent disease activity, including PIRA. Notably, a two-fold increase in baseline sNfL levels has been associated with up to a 2.3-fold higher risk of relapse during follow-up [64]. Furthermore, increased sNfL levels correlate with the emergence of Gd-enhancing lesions, typically peaking within approximately three months of their appearance [65]. While elevated sNfL levels are characteristic of periods of active inflammation and more severe MS phenotypes, a consistent decline in sNfL levels has been observed following the initiation of DMTs [66,67]. Moreover, treatment with monoclonal antibodies has been associated with a normalization of sNfL z-scores to levels comparable to those observed in controls without MS, an effect also observed with other DMTs, albeit to a lesser extent [68^▪^,^69^]. A recent international consensus has proposed that the absence of a decline in sNfL levels within 6 months following DMT initiation may signal suboptimal therapeutic response and warrant consideration of treatment escalation (Table 2) [70].

#### Glial fibrillary acidic protein

GFAP is an intermediate filament protein predominantly expressed in astrocytes, whose upregulation is a hallmark of reactive astrogliosis. When excessive, this process may culminate in astroglial scarring, increasingly recognized as a key contributor to disease progression in MS [71,72]. Emerging evidence indicates that GFAP demonstrates superior accuracy to sNfL in distinguishing PIRA, thereby underscoring the role of compartmentalized CNS inflammation as a principal driver of smoldering MS [72]. Notably, in contrast to sNfL, GFAP levels do not rise during episodes of acute inflammation, nor do they appear to be modulated by currently available DMT [73]. Thus, the concurrent evaluation of GFAP and sNfL may yield valuable insights for distinguishing RAW from PIRA, thereby providing a more nuanced framework for monitoring the MS course [72,74,75^▪^,^76^]. It appears that GFAP z-scores measured six months following DMT initiation, once inflammatory activity has been largely mitigated, are more closely associated with subsequent disability accrual, as reflected by inner retinal layer thinning, a well-established surrogate of neuroaxonal loss [77]. Nevertheless, serum GFAP concentrations display only a low-to-moderate correlation with corresponding CSF levels, which may constrain their applicability in routine clinical practice [78].

However, despite their growing promise, fluid biomarkers are not yet broadly applicable in real-world clinical practice due to limitations in cost-time efficiency, restricted availability of standardized and affordable assays, limited inter-assay comparability, and uncertainties regarding their clinical interpretability. At present, single measurements offer limited diagnostic value; instead, meaningful clinical use appears to require longitudinal assessment at approximately 3–6-month intervals, with results adjusted for age, body mass index and, in the case of GFAP, also sex, typically through percentiles or *z*-score normalization. While blood sampling itself is straightforward and can be completed within minutes by trained personnel, laboratory processing and analysis remain resource-intensive, and the establishment of robust prognostic thresholds capable of reliably guiding clinical decision-making remains an ongoing challenge. At present, there remains insufficient evidence to demonstrate that these biomarkers provide independent, additive predictive value beyond established clinical and radiographic response measures (e.g., NEDA). Consequently, despite their promising potential, current data do not yet support the routine implementation of fluid biomarkers in standard clinical practice (Table 2).

## NO EVIDENCE OF DISEASE ACTIVITY

Beyond single parameters, composite measures integrating clinical and paraclinical biomarkers have been developed to provide a more holistic assessment of treatment response [4]. Among these, the most widely adopted construct is No Evidence of Disease Activity (NEDA), most commonly operationalized as its three-component formulation, NEDA-3. NEDA-3 is fulfilled when a patient experiences no relapses, no confirmed disability progression, and no new or active MRI lesions during an observation period (Fig. 1). This composite benchmark has become a prominent therapeutic target in clinical trials and increasingly in routine practice, providing a pragmatic framework for gauging anti-inflammatory treatment efficacy [11]. Patients achieving NEDA-3 within the first one to two years of therapy generally exhibit more favorable short-term outcomes compared with those in whom DMT fails to achieve NEDA-3 [79,80]. Trials of high-efficacy DMTs consistently demonstrate higher proportions of individuals attaining NEDA-3, and NEDA status has been associated with a reduced risk of subsequent disability accumulation.

Nevertheless, it is now recognized that NEDA-3, although stringent, remains an incomplete descriptor of disease control. By focusing primarily on overt inflammatory activity, NEDA-3 may overlook ongoing subclinical neurodegenerative processes that ultimately drive disability. Patients receiving high-efficacy therapies frequently achieve NEDA-3 due to substantial suppression of relapses and MRI lesion activity, yet may continue to accrue damage through smoldering pathology, processes increasingly implicated in disability progression even in early relapsing disease [5]. In a long-term observational study, approximately one-quarter of individuals who met NEDA-3 at 2 years subsequently developed confirmed disability worsening, underscoring that “no evidence of disease activity” on conventional metrics does not necessarily ensure absence of disease progression [79]. Consistent with this, a recent pooled analysis of early RMS trial cohorts receiving high-efficacy therapy demonstrated that the vast majority of disability accumulation (78–89%) was attributable to PIRA [13].

To improve the sensitivity of composite measures for capturing neurodegenerative processes, several groups have proposed extending the NEDA framework with additional biomarkers. The most widely discussed extension, NEDA-4, incorporates brain volume loss (atrophy) alongside the traditional triad, reflecting the contribution of neuroaxonal injury to long-term outcomes, as discussed earlier. Other investigators have proposed integrating fluid-based biomarkers, most notably sNfL or GFAP, which show promise as indicators of neuronal and astroglial injury, respectively. However, current evidence remains insufficient to support routine clinical implementation of these biomarkers within NEDA formulations [76]. Similarly, NEPAD has been proposed to address more subtle disease progression in PMS and is increasingly used as a global endpoint in PMS trials, although its role as a routine clinical treatment target remains to be defined [34].

## CONCLUSION

In summary, the present work consolidates a growing body of evidence demonstrating that multimodal biomarkers, including clinical measurements, MRI and OCT parameters, as well as fluid biomarkers, offer complementary and biologically meaningful insights into the complex interplay between neuroinflammatory and neurodegenerative processes in MS. By incorporating methodological refinements such as re-baseline MRI and OCT, and *z*-scores of fluid biomarkers, we highlight how rigorous biomarker standardization can substantially enhance interpretability, reduce confounding influences, and improve the detection of treatment-specific effects.

Taken together, these findings support a continuous evolution toward a more comprehensive, multimodal approach to disease monitoring, one that extends beyond relapse counts and MRI lesion activity to more fully encompass the continuous spectrum of neuroaxonal injury. As biomarker standardization advances and longitudinal datasets mature, the incorporation of OCT and fluid biomarkers into routine clinical practice holds promise for refining therapeutic decision-making, enabling earlier detection of progression, and ultimately improving long-term outcomes for people with MS. Further prospective studies will be essential to validate these approaches, optimize thresholds for clinical interpretation, and define their utility across diverse patient populations and treatment contexts.

Notably, the majority of current studies continue to stratify treatment responses according to traditional MS phenotypes, such as RMS vs. SPMS or PPMS. However, the field is increasingly moving toward a unified conceptualization of MS, emphasizing shared underlying pathological mechanisms, which advocates for the development of integrated outcome assessments that transcend conventional subtype boundaries.

## Acknowledgements

*Figure 1* *was created in BioRender.com and exported under a BioRender publication license (Föttinger, F. [2026];* *https://BioRender.com/v800uxf**).*

### Financial support and sponsorship

*None.*

### Conflicts of interest

*Fabian Föttinger has participated in meetings sponsored by, received speaker honoraria or travel funding from Novartis, Biogen, Merck and Neuraxpharm.*

*Nik Krajnc has participated in meetings sponsored by, received speaker honoraria or travel funding from Alexion, BMS/Celgene, Janssen-Cilag, Merck, Neuraxpharm, Novartis, Roche and Sanofi-Genzyme and held a grant for a Multiple Sclerosis Clinical Training Fellowship Programme from the European Committee for Treatment and Research in Multiple Sclerosis (ECTRIMS).*

*Gabriel Bsteh has participated in meetings sponsored by, received speaker honoraria or travel funding from Biogen, BMS, Heidelberg Engineering, Janssen, Lilly, Medwhizz, Merck, Neuraxpharm, Novartis, Roche, Sanofi, Teva and Zeiss, and received honoraria for consulting Adivo Associates, Biogen, BMS, Janssen, Merck, Novartis, Roche, Sanofi and Teva. He has received unrestricted research grants from BMS and Novartis. He serves on the Executive Committee of the European Committee for Treatment and Research in Multiple Sclerosis (ECTRIMS) and the Board of Directors of the International Multiple Sclerosis VisualSystem Consortium (IMSVISUAL).*

## REFERENCES AND RECOMMENDED READING

Papers of particular interest, published within the annual period of review, have been highlighted as:

▪ of special interest
▪▪ of outstanding interest
